# Evaluation of COVID-19 cases treated in the intensive care unit in a coastal city hospital during the pandemic

**DOI:** 10.1590/1414-431X2024e14301

**Published:** 2024-11-04

**Authors:** P.H.A. Klauss, E.M.B. Hi, C.C.R. Bianchi, A.U. Ruiz, M.F.C.B. de Barros, B.M. da Silva, T.L. Moretto, F.G. Soriano, R. Curi, M.C.C. Machado, R.B. Gritte

**Affiliations:** 1 Laboratório de Emergências Clínicas, Departamento de Clínica Médica, Faculdade de Medicina, Universidade de São Paulo, São Paulo, SP, Brasil; 2 Centro Universitário Lusíada, Santos, SP, Brasil; 3 EU Business School, Munich, Bavaria, Germany; 4 Programa Interdisciplinar de Pós-Graduação em Ciências da Saúde, Universidade Cruzeiro do Sul, São Paulo, SP, Brasil; 5 Centro de Ensino, Instituto Butantan, São Paulo, SP, Brasil

**Keywords:** COVID-19, SARS-CoV-2, Platelets, D-dimer, CRP, INR

## Abstract

SARS-CoV-2 is a novel coronavirus that infects the respiratory tract and was the causing agent of COVID-19, declared a pandemic by the World Health Organization on March 11, 2020. Several studies have been carried out to understand the pathophysiology of the disease, immune reactions, and risk factors that could aggravate the condition and predict the prognosis of patients. Therefore, this study aimed to evaluate the most prevalent laboratory data of hospitalized patients associated with discharge or death. A survey was conducted utilizing the medical records of COVID-19 cases in patients treated in the intensive care unit of the Guilherme Álvaro Hospital in the seaside city of Santos, Brazil. We correlated the most important variables reported in the literature to provide a global comparison of the population affected by the virus in the Santos lowlands.

## Introduction

Coronavirus disease 2019 (COVID-19) is an infectious disease that primarily affects the respiratory tract and is caused by the SARS-CoV-2 coronavirus. It was first described in Wuhan, China and rapidly spread around the world. In March 2020, the World Health Organization (WHO) classified the outbreak as a pandemic. Many individuals who contract SARS-CoV-2 are asymptomatic, while others have a mild disease or no severe symptoms. On the other hand, there is a small group of patients (∼15%) in whom the disease progresses to a severe or critical condition with severe lung damage or even multiple organ failure (MOF) (1), which has led to a significant number of deaths worldwide.

MOF can involve any organ or system, with the lungs, cardiovascular system, kidneys, brain, liver, and blood coagulation system among the most affected (2). COVID-19, characterized by systemic thrombotic epitheliopathy, manifests as endothelial dysfunction, potentially serving as the connecting factor between the disease and MOF.

Most patients infected with SARS-CoV-2 often exhibit hyperinflammation, coagulation changes, and dysregulated immune responses resembling sepsis (3). Severe COVID-19 cases can trigger exacerbated inflammatory responses, and patients can progress to septic shock or MOF. This complex condition is closely linked to a deregulated immune response and excessive production of cytokines and chemokines, the so-called “cytokine storm” (4).

The clinical picture of patients hospitalized for complications of COVID-19 is usually characterized by blood tests and some biomarkers, such as platelet counts, international normalized ratio (INR), C-reactive protein (CRP), D-dimer, pro-calcitonin (PCT), and serum lactate (5). Recently, the WHO reported more than 78,000 new hospitalizations and 500 new intensive care unit (ICU) admissions due to COVID-19 from February to March 2024; however, there was an overall decrease of 34% in hospitalizations and 61% in ICUs admissions compared to 2023 (6).

This study surveyed COVID-19 patients admitted to the Guilherme Álvaro Hospital (HGA) ICU in Santos, State of São Paulo, Brazil, and compared them to other coastal and metropolitan cities worldwide. It also assessed the quality of life of surviving individuals using the SF-36 score.

## Material and Methods

### Study design

In this retrospective study, we surveyed COVID-19 cases treated in the HGA ICU and conducted a quantitative analysis of discharged patients and those who died. We also analyzed medical records to determine clinical and laboratory changes in both groups. The surviving patients (i.e., survivors) were contacted 1.5-2 years after hospital discharge and asked to complete a quality of life questionnaire (SF-36).

### Patients

Only patients who had a positive quantitative polymerase chain reaction (qPCR) test for SARS-CoV-2 (nasopharyngeal swab) and developed moderate symptoms (requiring hospitalization) or severe symptoms (requiring hospitalization and intubation) attended at the HGA ICU were selected. Data from patients hospitalized for reasons other than SARS-CoV-2 infection and/or medical records with incomplete or illegible data were excluded from the study.

Patients were divided into two groups: hospital discharge and death. The data from each group were compared to find clinical and laboratory differences that affected the outcome.

Data collection from the patients' medical records did not require a free and informed consent as it was retrospective data. However, volunteers who agreed to participate in the research and respond to the SF-36 questionnaire provided an informed consent. Patients were divided into mild and moderate/severe COVID-19 and filled out the questionnaire. The Ethics Committees of the participating institutions (Lusiada University Center (UNILUS), CAAE 51925221.5.0000.5436; Guilherme Alvaro Hospital, CAAE 51925221.5.3001.5448; and Cruzeiro do Sul University (UNICSUL) CAAE 51925221.5.3003.8084) approved this work.

### Medical records

From the medical records made available by the hospitals, we collected information such as the participant's gender and age, comorbidities (e.g., diabetes, hypertension, obesity, liver disease, nephropathy, chronic obstructive pulmonary disease, immunodeficiency, and/or neurological disease), length of stay, respiratory failure requiring intubation, and the outcome (discharge or death).

The laboratory tests analyzed in the medical records included the coagulation tests, platelet counts, and prothrombin activity time (PAT), INR, D-dimer, and CRP levels.

We collected 493 medical records from the HGA ICU, which was registered as a COVID-19 ICU from April 2020 to November 2021. After an initial screening, 126 records were excluded because they did not contain a diagnosis of SARS-CoV-2 infection confirmed by laboratory testing.

### SF-36 score

The SF-36 is a multidimensional questionnaire consisting of 36 items grouped into eight scales or domains: functional capacity (FP), physical aspects (PR), pain (BP), general health (GH), vitality (VT), social aspects (SF), emotional aspects (RE), and mental health (MH), assessed by 35 questions and an additional question comparing the current health status with that of one year ago (7). Its final score ranges from 0 to 100, with zero corresponding to the worst and 100 corresponding to the best general state of health.

Participants had access to the questionnaire electronically via Google Forms. The questionnaire and the way in which the results were calculated and interpreted are included in the supplementary material.

## Results

The medical records of 367 patients were used in this study, of which 194 were male and 173 were female. The average age was 54 years, with a minimum and a maximum of 17 and 91. In addition, the average length of stay was 13.5 days, with the shortest time being 1 day and the longest being 167 days (Table 1).

**Table 1:** Characteristics of the study population (n=367).

	Female	Male
Total population, n (%)	173 (47.14%)	194 (52.86%)
Age		
Mean (±SD)	54.5 (±14.5)	54.5 (±14.6)
Median (min-max)	57.0 (17-83)	55.0 (18-91)
Most common comorbidities, n (%)		
Hypertension	91 (52.60%)	105 (54.12%)
Diabetes mellitus	47 (27.17%)	63 (32.47%)
Obesity	64 (36.99%)	64 (32.99%)
Length of stay (days)		
Mean (±SD)	13.0 (±9.2)	14.1 (±17.9)
Median (min-max)	10.5 (1-59)	9 (1-167)
Case resolution, n (%)		
Survivors	105 (60.69%)	140 (72.16%)
Non-survivors	68 (39.31%)	54 (27.84%)

The hospital is a reference institution for severe cases in Santos and has worked almost exclusively with COVID-19 patients referred by the Brazilian Unified Health System (SUS). The number of infected patients treated at the hospital was much higher than the number in this study, because a change in the hospital's ICU service and records during the pandemic resulted in a substantial loss of data related to the unit's records.

The medical records collected showed that patients came from various cities in the state of São Paulo, with the highest frequencies coming from the small coastal towns Itanhaém (14.45%), São Vicente (12.72%), and Peruíbe (11.56%) (Table 2).

**Table 2:** Origin of COVID-19 patients seen at Guilherme Álvaro Hospital (Santos).

City	Hospitalized (%)
Bertioga	4.05%
Cubatão	10.98%
Guarujá	8.67%
Iguape	1.16%
Itanhaém	14.45%
Juquiá	0.87%
Mongaguá	10.69%
Peruíbe	11.56%
Praia Grande	4.62%
Registro	2.31%
Santos	8.96%
São Paulo	4.05%
São Vincente	12.72%
Others	4.91%

**Figure 1:**
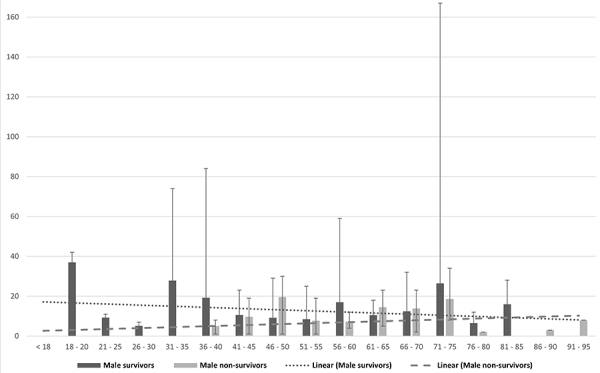
Length of stay (days) of male patients who were discharged or died. The dotted line indicates a downward trend in discharges, while the dashed line indicates a downward trend in deaths with increasing age. Data are reported as means±SD.

**Figure 2:**
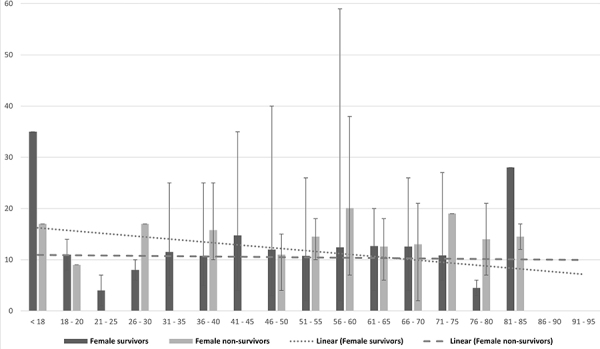
Length of stay of female patients who were discharged or died. The dotted line indicates a downward trend in discharges, while the dashed line indicates an upward trend in deaths with increasing age. Data are reported as means±SD.

When analyzed by gender, age was associated with length of stay. There was a tendency of a shorter length of stay with increasing age for discharged male patients, while in the case of deaths, the older the patient, the longer the length of hospitalization. In women, discharged cases were similar to men, but death cases showed no difference with age or length of stay (Figures 1 and 2).

**Figure 3:**
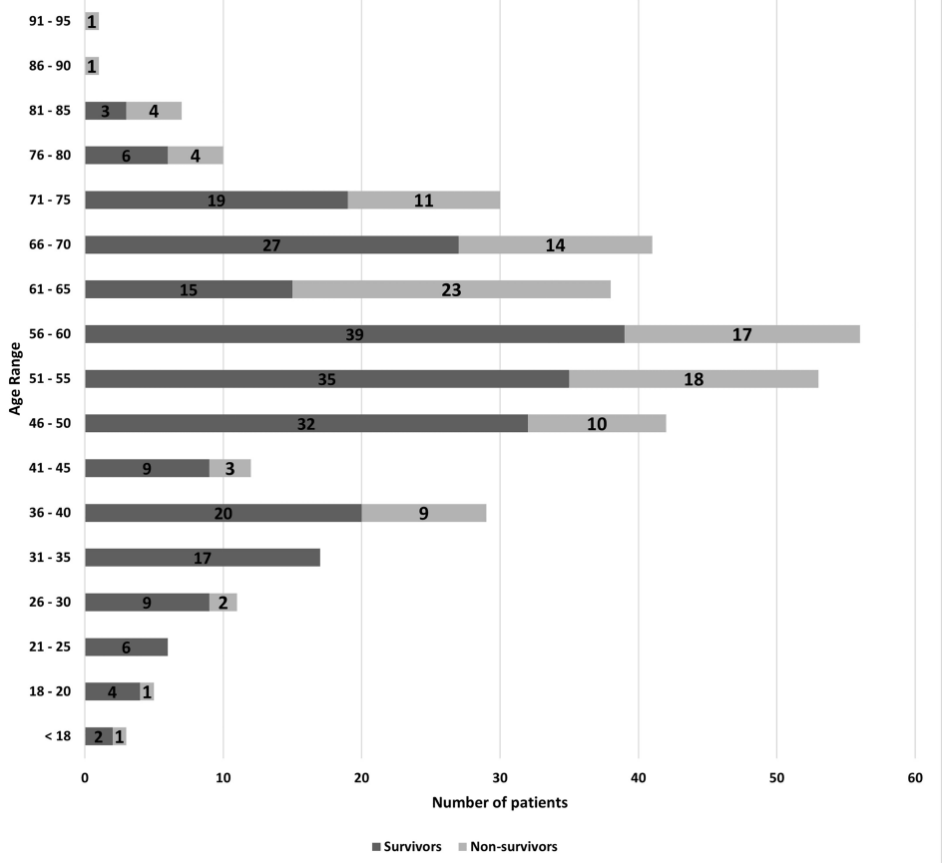
Relationship between the number of discharges and deaths separated by age group.

In total, 245 patients were discharged and 122 died. With this data, a tabulation was made using the number of individuals and their respective age groups, considering discharges and deaths (Figure 3). The age range in which most deaths occurred was between 61 and 85 years.

**Figure 4:**
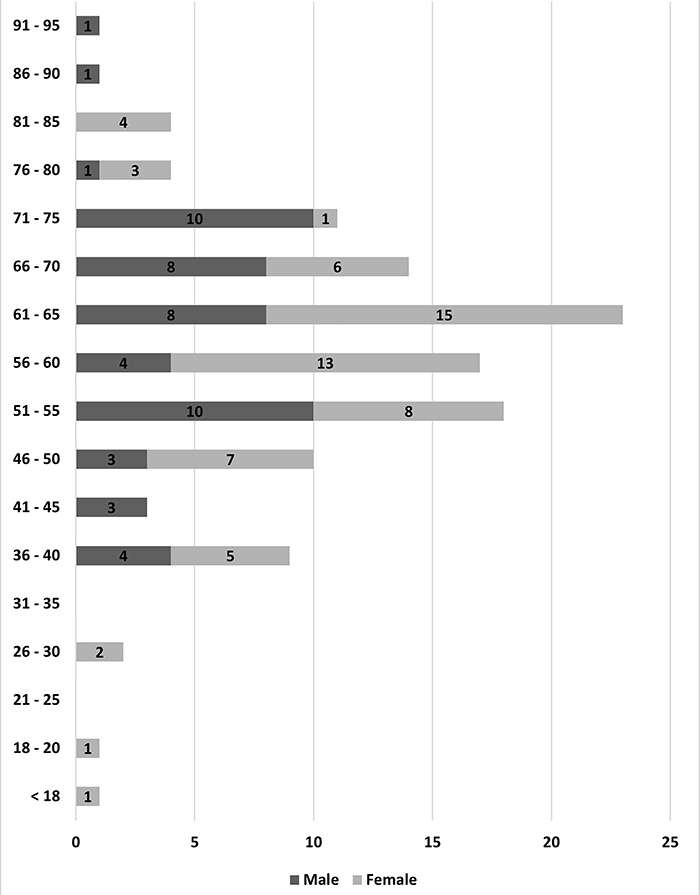
Number of deaths from COVID-19 by sex and age group.

Most deaths in males occurred in the 51-75 age group, with a predominance in the 61-75 group. In contrast, there were more female deaths than male deaths. Moreover, deaths among female patients were distributed across almost all age groups, with a predominance in the 56-65 group (Figure 4).

The comorbidities of the 367 patients included: systemic arterial hypertension (SAH) (53%), obesity (35%), diabetes mellitus (DM) (30%), chronic obstructive pulmonary disease (7%), neurological diseases (4%), nephropathy (3%), hepatopathy (1%), and immunodeficiency (1%). Considering the three most prevalent comorbidities - SAH, DM, and obesity - a combinatorial analysis was conducted to establish a relationship between them and the number of discharges and deaths (Table 3).

**Table 3:** Main comorbidities of survivors and non-survivors of COVID-19.

Comorbidities	Survivors	Non-survivors
Total population n (%)	173 (47.14%)	194 (52.86%)
SAH only	39	10
DM only	10	7
Obesity only	10	16
SAH + DM	31	18
SAH + obesity	43	15
DM + obesity	1	3
SAH + DM + obesity	23	17

SAH: systemic arterial hypertension; DM: diabetes mellitus.

**Figure 5:**
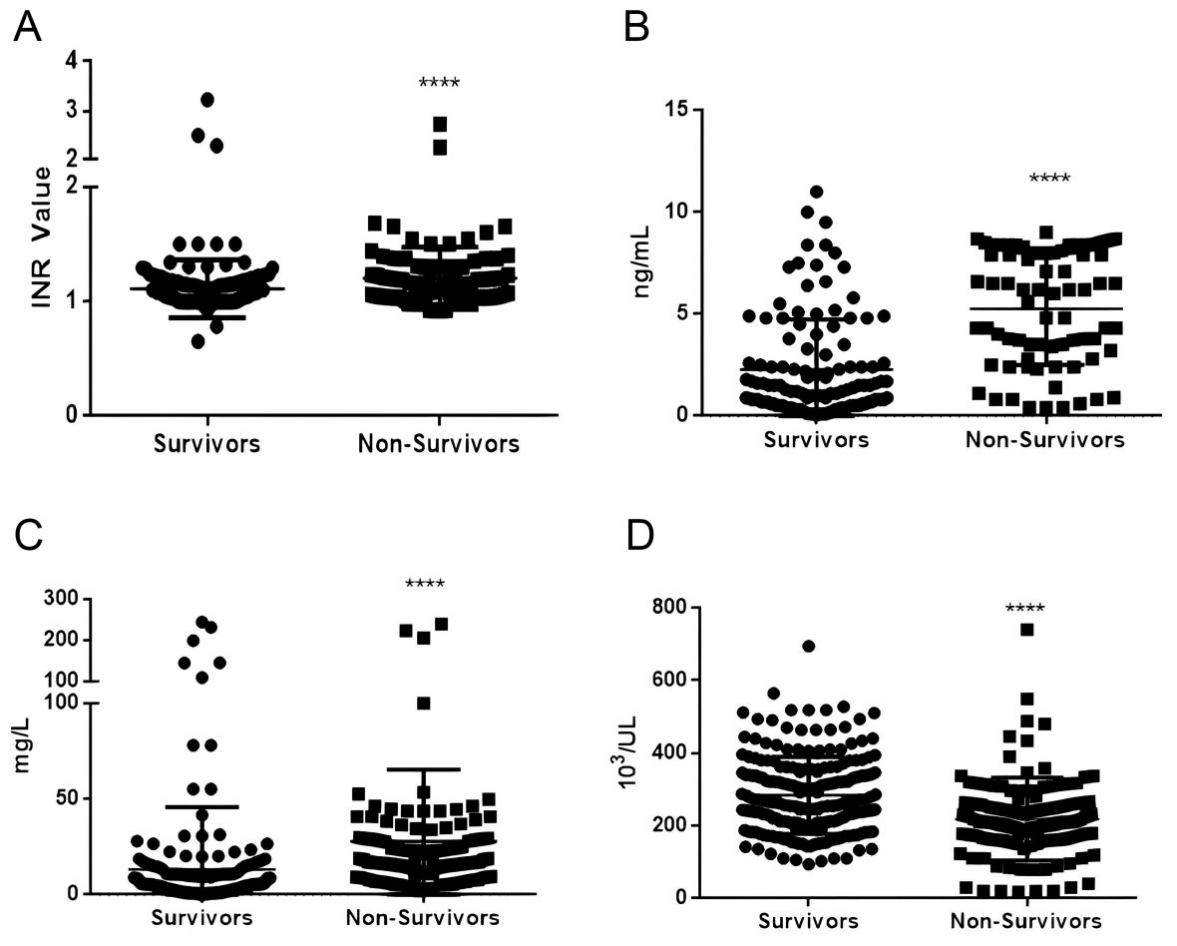
Blood tests and biomarkers of discharged COVID-19 patients compared to the COVID-19 patients who died. **A**, International normalized ratio (INR); **B**, D-dimer; **C**, C-reactive protein; and **D**, Platelet counts. Data are reported as means±SD. ****P<0.001 (*t*-test).

Figure 5A shows the mean INR of each patient admitted to the ICU, divided into survivors and non-survivors. In survivors, mean INR was within the upper normal limit (1.1, according to UCFS Health). However, in death cases, the average was 1.2, which is statistically significant (P<0.0001).

Figure 5B shows the average D-dimer levels of patients admitted to the ICU, divided into survivors and non-survivors. The average for survivors was considerably higher than the limit values (<0.5, according to StatPearls). By looking at the average for non-survivors, there was a significant increase of about 2.26 higher than the average for survivors (P<0.0001).

Figure 5C shows the results of CRP values compared to survivors, with values much higher than normal (i.e., <0.5 mg/dL according to the HGA reference values, but 6 mg/dL was used in this study due to interferences such as hypertension and diabetes), with non-survivors having a very high average value, approximately 2.5 times higher than that of survivors (P-value <0.001).

Figure 5D shows the results of platelet counts in survivors, with the average within the standard (150-400 thousand/mm^3^, according to Mayo Medical Laboratories). Non-survivors had a slightly lower average (∼1.27 times), but within the reference values. However, when looking at the standard deviation (SD), some cases of non-survivors had platelet numbers much lower than the minimum limit (P<0.001).

Table 4 shows a comparison of age, platelet, CRP, D-dimer, and INR values between the surviving and deceased patients from this study and those reported in other Brazilian cities (São Paulo and Rio de Janeiro) and the city of New York, USA, Chicago, USA, and Wuhan, China (8-18). There was a significant difference between the mean ages of survivors of Santos and Wuhan, suggesting that the younger population had a better outcome and that the population of survivors in Wuhan had a higher survival rate than those of Santos. However, the mean was higher in Chicago than in Santos, suggesting that an older population can also experience a good outcome.

**Table 4:** Findings of surviving (S) and deceased (D) patients from this study (Santos) and those reported in other cities.

Cities Markers	Santos	São Paulo	Rio de Janeiro	New York	Chicago	Wuhan
Age (years) (S)	54.5 (±14.5)n=243	58.3 (±17.3)n=30,344 **8**	48.9 (±17.01)n=1808 **9**	54.9 (±18.21)n=768 **10**	66.5 (±7,04)*n=212 **11**	46 (±20.41)*n=1019 **12**
Age (years) (D)	67.16 (±4.55)n=119	67.1 (±5.48)n=22,540 **8**	68.02 (±14.31)n=92 **9**	69.49 (±17.40)*n=310 **10**	68.0 (±6,3)n= 101 **11**	76 (±13.78)*n=37 **12**
Platelets (1000/mm^3^) (S)	262 (±112.92)n=214	236 (±53.63)*n=60 **18**	233 (±127.41)n=4,512 **13**	193 (±69.63)*n=726 **10**	208 (±75.56)*n=212 **11**	220 (±73.6)*n=137 **14**
Platelets (1000/mm^3^) (D)	210 (±113.74)n=111	176 (±70.37)n=18 **18**	219 (±97.78)n=683 **13**	185 (±70.37)*n=282 **10**	210 (±91.85)n=101 **11**	165 (±90.37)*n=54 **14**
CRP (mg/dL) (S)	18.11 (±34.95)n=197	89.7 (±41.26)*n=31 **15**	48 (±62.44)n=4,512 **13**	79.3 (±78.89)*n=422 **10**	9.25 (±8.81)*n=212 **11**	12.3 (±16.22)*n=193 **16**
CRP (mg/dL) (D)	27.82 (±37.47)n=101	179.5 (±44.96)*n=24 **15**	93.2 (±99.11)n=683 **13**	162 (±141.04)*n=141 **10**	13.9 (±9.77)*n=101 **11**	44 (±45.41)n=20 **16**
D-dimer (µg/mL) (S)	3.41 (±2.95)n=120	0.96 (±0.29)*n=57 **18**	0.93 (±2.59)*n=4,512 **13**	0.93 (±0.76)*n=282 **10**	1.19 (±1.94)*n=212 **11**	0.6 (±0.52)*n=137 **14**
D-dimer (µg/mL) (D)	5.27 (±2.76)n=73	2.06 (±2.95)*N=18 **18**	1.8 (±3.04)*n=683 **13**	2.41 (±1.93)*n=117 **10**	2.44 (±5.07)*n=101 **11**	5.2 (±14.52)n=54 **14**
INR (S)	1.18 (±0.71)n=160	1.08 (±0.05)n=104 **17**	1.1 (±0.22)n=4,512 **13**	1.07 (±0.10)n=304 **10**	1.26 (±0.16)n=104 **17**	0.86 (±0.16)*n=137 **14**
INR (D)	1.20 (±0.27)n=89	NF	1.1 (±0.22)*n=683 **13**	1.14 (±0.21)*n=142 **10**	1.48 (±0.23)*n=38 **17**	0.93 (±0.19)*n=54 **14**

Data are reported as means and SD. *P<0.05 compared to Santos (*t*-test). NF: not found; INR: international normalized ratio. The study reference numbers are cited in bold type within parentheses.

Age of deceased was statistically different in Wuhan and New York. Wuhan showed a higher mean of age, suggesting that an older population was more affected by the disease than the younger population, which had a better outcome. The New York had the highest SD, suggesting that the age of affected citizens was sparser.

Platelet level of survivors was statistically different in São Paulo, New York, Chicago, and Wuhan. The average number of platelets (and SD) in these cities was lower than in Santos, even considering each city's population. On average, platelet level of survivors was lowest in New York, with some patients presenting lower counts than the reference. The three others had a value within the reference value for platelets.

The platelet level of the deceased was statistically different in Wuhan and New York. Wuhan had a lower mean, with the lowest overall platelet level compared to Santos. There was also a higher tendency to develop thrombocytopenia. New York presented a low mean platelet level, with one of the lowest SDs, suggesting a more even population.

The CRP of survivors was statistically different in São Paulo, New York, Chicago, and Wuhan. São Paulo had the highest average CRP value compared to the other cities. New York had the second highest mean value, but the SD was broad. On the other hand, Chicago had the lowest average CRP, suggesting a prevalence of attenuated inflammatory markers in survivors. Wuhan had a medium average, but large SD, indicating possible outliers (i.e., people with extraordinarily large or small values) that affects the mean.

São Paulo had the highest mean CRP of deceased compared to the other cities, suggesting a stronger presence of inflammation. New York had the second largest mean, but some values were higher than in São Paulo due to the broad SD. Chicago, similar to the CRP of survivors, had the lowest value, suggesting that the population of this city may not express high CRP, regardless of the outcome.

Regarding D-dimer of survivors, São Paulo had a medium average, still lower than Santos. Nevertheless, the SD suggested a more even population, with little variance. In Rio de Janeiro, the SD was higher than the mean, which indicates a more dispersed population with a wide range of values and no discernible pattern. New York had a lower mean and SD compared to Santos, suggesting a lower activity of plasmin (the enzyme that creates the D-dimer from fibrin). Chicago had a high mean value but still lower than Santos. However, it presented a high SD.

São Paulo had a lower mean D-dimer value for deceased with a high SD. Rio de Janeiro had the lowest average but a large SD. New York had a lower mean D-dimer value than Santos and a large SD. Chicago had a large average, and the SD was greater than in Santos.

Wuhan was the only city with a statistically lower value of INR of survivors, suggesting a lower coagulation time than the other cities. At some point, this characteristic could be beneficial for survival.

Finally, all the cities showed statistically different INR of deceased than in Santos. Rio de Janeiro, New York, and Wuhan also had lower average values than Santos, which could indicate faster coagulation activation. On the other hand, Chicago had the highest INR value. This result suggested an obstacle in coagulation activation in Chicago's population.

### SF-36 score

Eighty-two of the survivors were interviewed, of which 29 had a mild form of the disease (mild COVID-19 group) and 53 had a severe form (moderate/severe COVID-19 group).

Table 5 presents the mean scores of the eight domains for each group. The first domain, “Physical Functional Capacity”, consists of ten questions to evaluate the individual's capacity to perform common physical activities of daily living, such as walking, climbing stairs, and carrying heavy objects. The global mean for this domain in the mild COVID-19 group was 80 (±25.7), while in the moderate/severe COVID-19 group, it was 60.5 (±30.9) (P<0.01). According to the study by Laguardia et al. (19) applied to a healthy population before the pandemic, the standard value for this domain is 75.5. Thus, the mild COVID-19 group presented a score within the normal range for a healthy population, while the score for the moderate/severe COVID-19 group was slightly below average. Additionally, Villa e Vila et al. (20) applied the questionnaire during the pandemic and observed that the average for patients not infected by the disease was 93.3, which was much higher than in our groups.

**Table 5:** SF-36 scores of post-COVID-19 population compared to healthy populations.

Domain	Mild disease	Moderate/Severe disease	Healthy populationBefore COVID-19 19	Healthy populationDuring COVID-19 20
Functional Capacity	80.0	60.5	75.5	93.3
Physical aspects	57.5	45.4	77.5	74.2
Pain	70.4	55.7	76.7	74.0
General state of health	55.4	53.9	70.2	71.3
Vitality	48.0	44.4	71.9	54.0
Social aspects	67.2	63.9	83.9	65.9
Emotional aspects	40.0	45.7	81.7	49.4
Mental health	75.1	69.4	74.5	63.5

19: Laguardia et al. (https://doi.org/10.1590/S1415-790X2013000400009); 20: Villa e Vila et al. (https://doi.org/10.11606/issn.1679-9836.v101i6e-199108).

The second domain, “Limitations due to Physical Aspects”, assesses the impact of physical health problems on daily activities and work performance through four questions. The global mean for this domain in the mild COVID-19 group was 57.5 (±42.1), while in the moderate/severe COVID-19 group, it was 45.4 (±41.5). Although no difference was found between groups, both scored lower than the healthy population, which had an average of 77.5 before the pandemic (19) and 74.2 during the pandemic (20).

The third domain, “Pain”, assesses the intensity and frequency of pain experienced by the individual, including chronic pain, discomfort, and limitations due to pain. The global mean for this domain in the mild COVID-19 group was 70.4 (±19.4), while in the moderate/severe COVID-19 group, it was 55.7 (±26.3). A statistical difference (P<0.01) was found between the two groups in this domain. Before the pandemic, the average for the healthy population was 76.7, while during the pandemic, it was 74.0 (19,20), showing that both groups had scores below that of the healthy population.

The fourth domain, “General Health Status”, which consists of five questions, assesses the individual's overall perception of their health status, regardless of any specific condition. The global mean for this domain in the mild COVID-19 group was 55.4 (±14.9), while in the moderate/severe COVID-19 group, it was 53.9 (±19.5). The values did not differ between groups and were below the average of the healthy population (70.2 before the pandemic (19) and 71.3 during the pandemic (20).

The fifth domain, “Vitality”, has four questions to assess the individual's overall energy level. The global mean for this domain in the mild COVID-19 group was 48.0 (±24.0), while in the moderate/severe COVID-19 group, it was 44.4 (±23.6). The values did not differ between groups and both scores were below average scores for healthy individuals before the pandemic (71.9) (19) and during the pandemic (54.0) (20).

The sixth domain, “Social Aspects”, evaluates the participation and quality of the individual's social activities and interactions through two questions. The global mean for this domain in the mild COVID-19 group was 67.2 (±29.4), while in the moderate/severe COVID-19 group, it was 63.9 (±28.6). No statistical difference was found between the groups in this domain. According to Laguardia et al. (19), the score for the healthy population was 83.9, indicating that both groups were below that of the healthy population. However, according to Villa e Vila et al. (20), the score was 65.9, showing that the mild COVID-19 group was within the normal range, while the moderate/severe COVID-19 group was slightly below average. This difference between the two studies used for comparison may be due to the period in which the questionnaires were applied.

In the seventh domain, “Limitations due to Emotional Aspects”, composed of three questions, assesses how mental health problems affect daily activities and the individual's performance at work. The global mean for this domain in the mild COVID-19 group was 40.0 (±44.1), while in the moderate/severe COVID-19 group, it was 45.7 (±44.5). Although no statistical difference was found between groups, both presented scores below the average compared to the healthy population before (81.7) and during the pandemic (49.4) (19,20).

In the eighth domain, “Mental Health”, five questions assess the individual's ability to cope with emotional and mental health issues such as stress, anxiety, and depression. The global mean for this domain in the mild COVID-19 group was 75.1 (±23.2), while in the moderate/severe COVID-19 group it was 69.4 (±23.6). No statistical difference was found between groups. The score for a healthy population was 74.5, so the mild COVID-19 group had a similar value for this domain, while the moderate/severe COVID-19 group was below the average. However, during the pandemic, the score in this domain was 63.5, which was similar for both groups within the normal range obtained in a healthy population (19,20).

## Discussion

The main causes of death in our study were septic shock (55.74%), followed by acute respiratory failure (32.79%), pneumonia (32.79%), and acute renal failure (28.69%), along with a few cases involving acute myocardial infarction (3.28%). This result corroborated the characteristics of SARS-CoV-2 infection, which causes a septic condition in the most severe cases, a condition explained by the cytokine storm. Sepsis consists of two phases: hyperinflammatory and immunosuppressive. In the hyperinflammatory phase, the markers are CRP, interleukin (IL)-6, IL-1β, pro-calcitonin, and tumor necrosis factor alpha (TNF-α). These markers have also been reported to be elevated in cases of severe COVID-19 (4,21).

In severe cases, laboratory markers such as D-dimer, ferritin, and CRP show significant changes indicative of a cytokine storm (4). Some pro-inflammatory cytokines and chemokines, such as IL-6, IL-1, IL-18, and TNF-α, have been detected in high levels in patients with severe COVID-19 compared to non-severe cases (22). This condition, therefore, suggests that tissue damage, which can lead to organ failure and acute respiratory distress syndrome (ARDS) in SARS-CoV-2 patients, is primarily the result of the cytokine storm (23).

Obesity was the strongest risk factor for death (62.5%), followed by diabetes (41.1%) and hypertension (20.4%). Various studies carried out around the world have shown that the comorbidities that pose the greatest risk to patients are hypertension, diabetes, and obesity, our findings were consistent with this (12). Additionally, the study by Richardson et al. (24), with 5700 patients from New York, Long Island, and Westchester, who were hospitalized due to COVID-19, reported the presence of comorbidities such as hypertension (56.6%), obesity (41.7%), and diabetes (33.8%). Notably, all of these comorbidities have a direct or indirect relationship with the renin-angiotensin-aldosterone axis (25).

The high INR values may reflect a slower coagulation response after damage to the endothelium, which can result in minor bleeding or hemorrhages. Indeed, some of the complications reported were intracranial hemorrhage and disseminated intravascular coagulation (26).

In a systematic review of 30 studies from different countries, Zinellu et al. (27) showed that prolonged INR is associated with COVID-19 severity and mortality (P<0.001). The formation of thrombi over time can be considered one of the characteristics of severe COVID-19. Hypercoagulability is followed by a phase of disseminated intravascular coagulation (DIC), which is associated with pulmonary impairment and multiple organ disorder, which are detected by INR prolongation, thrombocytopenia, and increased D-dimer concentrations.

D-dimer values can indicate the activation of the coagulation system, resulting in deep vein thrombosis, pulmonary thromboembolism, or DIC, advanced age, post-surgery, major trauma, and inflammatory states. This situation occurs due to injuries and exposure of tissues to blood, which activates the coagulation system to reverse and eliminate the stimulus (28). In a systematic review of 13 studies, Vidali et al. (29) showed that a high elevation of the D-dimer is associated with severe COVID-19 and mortality (P=0.017). Increased D-dimer levels and hypercoagulation due to the cytokine storm might increase the risk of thrombus formation, worsening the patient's prognosis (23).

An increase in D-dimer levels can indicate the occurrence of DIC associated with pulmonary impairment and multiple organ disorder, which leads to an increase in coagulation and, over time, a delay in coagulation response, demonstrated not only by D-dimer levels but also by the elevated INR (28,30). In conjunction with IHD, the patient may develop ARDS, which further increases the state of hypercoagulability, thus further raising plasma D-dimer levels (30).

Elevated CRP levels can indicate inflammatory and/or infectious conditions, and increased cardiovascular risk. When macrophages are activated due to a stimulus, various cytokines are released, but IL-6 is of great importance since it targets hepatocytes and thus stimulates the production of CRP (30).

In a meta-analytical study using 16 studies, Zeng et al. (31) showed that elevated CRP is associated with COVID-19 severity and mortality (P<0.001). Other pro-inflammatory markers such as procalcitonin, IL-6, and erythrocyte sedimentation rate were positively correlated with COVID-19 severity. Another meta-analysis of 38 studies by Zinellu et al. (27) showed a strong positive association (P=0.048).

CRP may increase because of the cytokine storm. Viral activity in the body stimulates various immune cells to produce and secrete cytokines, generating a hyperinflammatory state. According to Lin (21), damage to the heart, liver, kidneys, and other organs, along with laboratory abnormalities such as D-dimer, CRP, and increased cytokines are similar to the sepsis condition caused by bacterial infections.

Reduced platelet counts (thrombocytopenia) can occur in various conditions, such as bone marrow diseases, hereditary diseases such as Wiskott-Aldrich syndrome, some viral infections, and autoimmune diseases (32). However, the greatest risk of thrombocytopenia is bleeding and hemorrhage, as the clot formation process is affected, slowing down or generating a very small “stagnation” but unable to prevent the extravasation of blood to the tissue and vice versa.

In a meta-analysis using nine studies, Lippi et al. (33) found that thrombocytopenia is associated with an increased risk of severity and mortality in patients with COVID-19, with an OR of 5.13 (P<0.001). In another study by Elbadawy et al. (30), the cytokine level was measured and correlated with other laboratory tests, one of which was TNF-α, which had a negative correlation (P<0.05) with platelet level.

In the study conducted by Guirado et al. (34), in which a questionnaire was applied before and during the pandemic, it was found that the pandemic did not alter the quality of life of study participants. This result shows that the pandemic began to significantly affect the population's quality of life over the years, as evidenced in the present study.

Poudel et al. (35) evaluated 12 studies using various questionnaires, including five studies that used the SF-36, and found that long COVID-19 can considerably affect the quality of life of individuals due to worsening health conditions.

According to da Silva and de Souza (36), fatigue and muscle changes can also occur due to administering neuromuscular blockers and the length of immobility in the ICU. In the study by Williams et al. (37), it is mentioned that fatigue in COVID-19 is associated with high levels of persistent inflammatory cytokines, a result of the cytokine storm from the acute phase of the virus infection. Another survey by Disser et al. (38) strengthens that patients may continue to experience symptoms of fatigue and muscle weakness after infection due to the pro-inflammatory effects of the viral infection and physical deconditioning during the recovery period. This situation could account for the low score in the functional capacity domain obtained in this study.

In a report by Offord (39), the author suggests that inflammation caused by the virus triggers a decrease in serotonin levels, which explains concentration and memory problems after the illness. Tests on mice confirmed that SARS-CoV-2 infection decreased serotonin blood levels. This result could also justify the low score in the emotional aspects domain.

It is important to mention that social isolation and the numerous changes in human behavior resulting from quarantine may have influenced the population's quality of life, even in individuals who did not develop the disease. We used a study that applied the same questionnaire to healthy individuals before and after the pandemic as a reference of normality to exclude possible bias caused by quarantine and isolation. In summary, there was a significant difference in the functional capacity and pain domains, which indicates common physical activities such as walking, climbing stairs, carrying weight, and intensity and frequency of pain.

**Figure 6:**
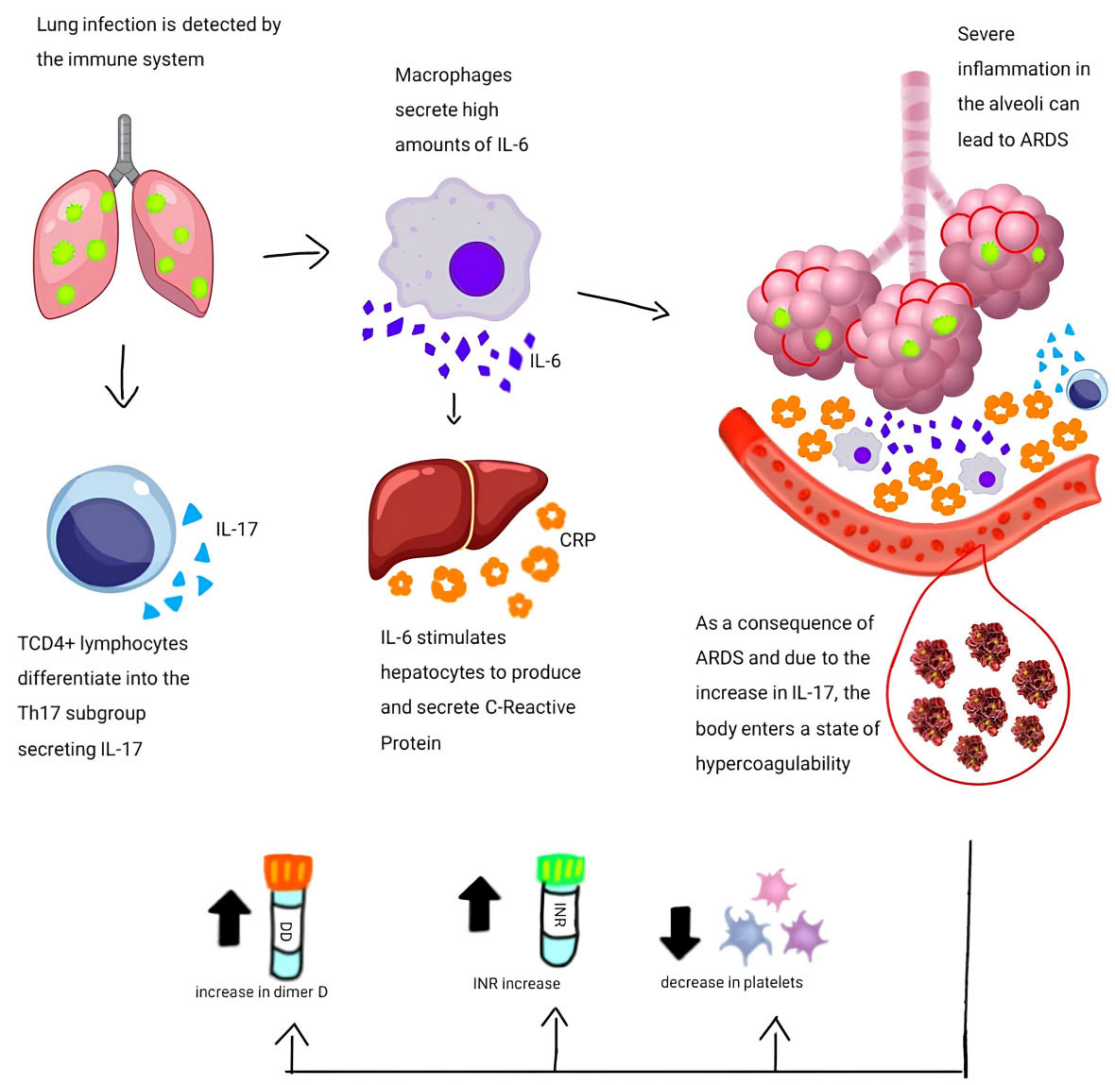
Immune-mediated mechanism and alterations in C-reactive protein (CRP), D-dimer, international normalized ratio (INR), and platelet levels in COVID-19 patients. ARDS: acute respiratory distress syndrome; IL: interleukin.

Figure 6 shows the interplay between the biological markers used in this study and disease events due to SARS-CoV-2 infection. After infection, the virus uses type 2 pneumocytes to replicate, activating the immune system. The cells of the innate immunity system, the first defense cells, begin to secrete cytokines to activate other inflammatory markers, including macrophages secreting IL-6 and stimulating the production of CRP (4). Over time, the acquired immunity begins to take hold, and T-helper lymphocytes differentiate into Th17, secreting IL-17.

The combination of these cytokines results in severe lung inflammation, which can lead to ARDS, which, together with IL-17, causes a state of local hypercoagulability (22). However, the excess of these cytokines, generated by the cytokine storm, can have a systemic effect, reducing the number of platelets and increasing clotting time, demonstrated by the increase in INR (due to the low platelet levels) and an increase in D-dimer (due to the high breakdown of thrombi formed) (24,28).

### Conclusion

The COVID-19 pandemic had a devastating impact globally, claiming over 14 million lives and overwhelming healthcare systems, leading to the establishment of makeshift facilities to cope with the surge in patients. Despite initial challenges, the scientific community united in a concerted effort to understand and combat the virus, conducting crucial research into risk factors such as age, gender, and comorbidities, while laboratory tests during ICU stays provided invaluable insights into disease progression and treatment strategies. As the acute phase of COVID-19 continues to be studied, there is a growing urgency to investigate survivors' long-term physiological, histological, biochemical, and immunological consequences, even as the pandemic evolves into a new phase.
